# Infant Complementary Feeding Methods and Subsequent Occurrence of Food Neophobia—A Cross-Sectional Study of Polish Children Aged 2–7 Years

**DOI:** 10.3390/nu15214590

**Published:** 2023-10-28

**Authors:** Agnieszka Białek-Dratwa, Oskar Kowalski

**Affiliations:** 1 Department of Human Nutrition, Department of Dietetics, Faculty of Public Health in Bytom, Medical University of Silesia in Katowice, ul. Jordana 19, 41-808 Zabrze, Poland; 2 Department of Cardiology, Congenital Heart Diseases and Electrotherapy, Silesian Center for Heart Diseases, 41-800 Zabrze, Poland

**Keywords:** child nutrition, complementary feeding, feeding disorders, food neophobia

## Abstract

Food neophobia is standard behaviour in child development. It is a complex process and occurs to varying degrees. The symptoms of neophobia can be variable depending on the individual. Food neophobia is a fear of new foods, whereby difficulties in eating and trying unfamiliar foods follow. It is one of the more vital determinants of the number of meals consumed at a young age. Such a process is not a disorder in itself but can lead to one. The highest severity of neophobia occurs between the ages of two and six, but in some children, it lasts beyond age 6. This study aimed to assess the prevalence of food neophobia among children aged 2–7 years, taking into account the method of complementary feeding, the length of breastfeeding, exclusive breastfeeding, the period of introduction of complementary foods, and the use of the BLW method during the period of dietary expansion. Materials and methods: The study used an anonymous survey questionnaire consisting of five parts as the research tool. The first part of the questionnaire was a metric and concerned the socio-demographic data of the parent/guardian and their child. A standardised questionnaire assessing food neophobia among children was used to assess food neophobia: the Food Neophobia Scale—Children (FNSC). Results: In the study group, 171 children (29.23%) had a low risk of food neophobia according to the FNSC, 182 children (31.11%) had a medium risk of neophobia, and 232 children (39.66%) had a high risk of neophobia. A correlation was observed between the age and the risk of food neophobia (*p* = 0.0002). Statistically significant differences were found between children aged 2 and 4 (*p* = 0.003) and children aged 2 and 5 years (*p* = 0.049). We observed no correlation between gagging (*p* = 0.88557), choking (*p* = 0.17597), and needing medical intervention (*p* = 0.61427) and the risk of associated neophobia. Conclusion: In the study group of children, the highest risk of food neophobia was characterized by children aged 4, 5, and 7 years. The length of breastfeeding and exclusive breastfeeding did not affect the risk of food neophobia. In the month in which complementary feeding (CF) was introduced, the children were fed using the baby-led weaning method (BLW method), and introducing puree and puree with lump food into the children’s diet also did not affect the risk of food neophobia. It was shown, however, that children whose mothers observed difficulties during CF and whose children had a vomiting reflex and spat food out of their mouths during CF were more likely to develop food neophobia at the preschool age.

## 1. Introduction

Proper nutrition in the first months of a child’s life is primarily based on meeting the energy and essential nutrient requirements to ensure the infant’s normal physical and psychomotor development. Nutrition during this period is also essential for so-called metabolic programming [1].

Failure during complementary feeding (CF) or sudden cessation of eating various foods makes parents increasingly concerned about their child’s monotonous diet and mealtime behaviour. The eating behaviour of infants and young children is highly individual and depends on many factors, including physiological development stage temperament [2]. In the case of infants, significant influences on feeding include the child’s readiness to accept solid foods, acquired oral–motor skills, and taste preferences [3]. Since 2002, the WHO has recommended exclusively breastfeeding infants for the first 6 months of life to optimize their growth, development, and health [4,5], while the European Society for Pediatric Gastroenterology, Hepatology, and Nutrition (ESPGHAN) recommends that “complementary foods should not be introduced earlier than 4 months of age, but should not be delayed beyond 6 months” [6]. In most children, introducing complementary foods into the diet is advisable around 6 months, when infants’ nutrient requirements increase [7]. Supplemental foods are designed to meet the need for protein, additional energy, fat-soluble vitamins (A, D, E), iron, zinc, and trace elements [1].

Parents must understand that their children are learning and beginning to develop taste preferences, especially during the complementary feeding and early school years. During infancy, babies fed breast milk and milk formula are mainly exposed to sweet tastes. Therefore, with CF, they are only just beginning to explore new tastes: sweet, bitter, sour. During this period, babies begin to acquire the ability to accept solid foods.

For adults, feeding is an easy activity. However, it is a very complex process that requires the interconnection of ages of systems and the coordination of the central nervous system with the peripheral nervous system. It is essential to have a properly functioning oropharyngeal mechanism and a properly and efficiently functioning respiratory system during the feeding process. A properly functioning feeding process must also include a properly functioning digestive system. Coordination of the muscles and structures in the oral cavity and the cranial and spinal nerves is essential for proper feeding. Coordination of the lips, jaw, cheeks, tongue, and soft palate is necessary for proper feeding. It is also essential to coordinate the sucking function to prevent aspiration of swallowed food into the airways. In the case of older children, they must be able to coordinate taking in solid food, breathing, and swallowing. Almost all experts agree on the possibility of introducing solid foods when the child is about 6 months old, sits stably with support, can control head movements and does not reflexively push out products in the mouth with the tongue [1,6,8,9].

The ability to take in solid foods matures in most infants between 17 and 26 weeks of age. Infants gain the ability to sit with support and gain neuromuscular maturity that allows them to control their head and neck movements and eat from a spoon. There is then an atrophy of the reflex, the removal of foreign bodies from the mouth, which facilitates feeding with food other than liquid. They are designed to meet the need for protein, additional energy, fat-soluble vitamins (A, D, E), iron, zinc, and trace elements [1,6,7]. 

These complex processes in young children whom a parent or caregiver is still feeding also require properly coordinated interactions between them. Otherwise, feeding problems can arise [1,6].

The baby-led weaning (BLW) method is one of the complementary feeding methods that is becoming increasingly well-known and more widely used. This method can be introduced when a baby becomes physically ready to eat on his or her own to effectively supplement his or her diet, which has hitherto been based on breast milk or formula mix. Using the BLW method, the child manages the self-feeding process using his or her skills and instincts. Brown and Lee define BLW as “a procedure in which the infant feeds itself, and feeding by the parent or administering liquid purees may occur occasionally, accounting for up to 10% of total feeding time”. The first solid foods introduced into an infant’s daily diet are called complementary foods because they are not intended to replace breast milk or formula milk but should complement them [10,11,12,13,14].

The terminology of problems related to feeding, eating, and nutrition is quite extensive. Food selectivity, food neophobia, picky eating, food aversion, and food avoidance/restriction all have one common denominator for parents: “my child does not eat” [9,15]. Under this term, however, there is much more than the mere fact of not taking in (in the opinion of parents) an adequate amount of food, and dietitians and paediatricians very often hear this in their practice.

Food neophobia is one of the eating behaviours most commonly observed in children, which causes great anxiety among parents [16]. This behaviour is characterised by the child rejecting new or unfamiliar foods. Neophobic children reject new foods visually (e.g., a new shape of pasta or a differently sliced vegetable) and in terms of taste (the food they are unfamiliar with). Neophobic behaviour can occur to a small extent as early as the first year of life but most often intensifies between 18 and 24 months. The period around two years of age is associated with increased mobility of the child and the occurrence and expression of their autonomy, which children can manifest during meals. It has been observed that the period of food neophobia overlaps with the time when the child’s growth and development rate start to slow down. The stage of food neophobia is one in the child’s life that should eventually resolve spontaneously [16,17,18,19,20].

Food neophobia is a natural stage of child development. It does not require treatment or therapy if it runs its natural course, and the child usually grows. It does, however, require nutritional education by the parent. Neophobia can be associated with various factors, such as personality traits, age, gender, or family socioeconomic status, and its severity may have a genetic basis [21]. With the development of neophobia comes reduced dietary variety and a decrease in the foods the child accepts. Young children often evaluate meals by observation. If they are served a portion of food on their plate in a different form than what they are already familiar with, it is often rejected as a new, unfamiliar food. Such aversion can occur when food is served in a different colour, shape, or form than the child expects, when an ingredient touches another food on the plate (e.g., potatoes coloured red because they are placed next to beets), or when it is in a different package [14,22]. 

According to the biopsychosocial approach, it is essential to understand the biological basis of the child’s behaviour and then link this to social and psychological processes. This will help to understand the mechanisms and processes of eating behaviour and its consequences [23]. Considering the biological basis of food neophobia, it can be observed that specific biological characteristics, such as heredity, temperament, age, gender, birth weight, and sensory acuity, can be linked to fussy eating behaviour in children [23]. In observing studies on picky eating, it has been observed that temperament, together with emotionality [24,25,26], traits such as fear, shyness, and impulsivity, may determine this trait [26,27,28]. Studies have also shown that sensory traits, such as taste/smell, mouth/finger touch, and general sensory sensitivity, are associated with children’s food choosiness [29] and food neophobia [30,31]. Negative emotionality (fearfulness or withdrawal) is the most salient aspect of temperament in eating neophobia [27].

Children’s eating behaviour, such as frowning or food refusal, can be defined as a pattern of behaviour that includes food choice and consumption [32,33]. A study by Moding K. et al. showed that children with low temperament may be at higher risk of developing food neophobia than their peers. Certain parental behaviours may reinforce tendencies towards neophobic behaviour, such as exerting pressure on eating practices [27].

Picky eating is also observed when children are tired, while other children’s picky eating behaviour may be for most of the day [33]. Biopsychosocial models identify additional elements of eating behaviour among children. These include, for example, sensory exposure, including visual, tactile, and gustatory exposure; behavioural and biological responses to visual, tactile, and gustatory experiences; positive and negative food experiences; and the desire to taste. These models identify cultural determinants and practices regarding food and eating by children. These models help conceptualise eating behaviours, such as states and traits, as developmental and context-dependent behaviours. Another area related to measuring eating behaviour among children originates from neuropsychology and health neuroscience and may be helpful in conceptualising children’s eating behaviour [33].

This manuscript continues to present the study’s results: “Food neophobia among infants and children”, conducted from January to March 2023. The study consisted of five components: metric data and a medically based interview, an assessment of feeding/nutrition during the neonatal–infant period and complementary feeding, a current assessment of children’s feeding and diet, and an assessment of the prevalence of nutritional neophobia based on a questionnaire (Food Neophobia Scale—Children (FNSC)), and an assessment of feeding difficulties based on The Montreal Children’s Hospital Pediatric Feeding Program (MCH-FS).

The first part of the results of this study has been published [34] and the results in this publication looked at the prevalence of feeding difficulties among children aged 2–7 years; the Montreal Children’s Hospital Pediatric Feeding Program (MCH-FS) was used as the survey tool. Moreover, the results are available via the following link: https://www.mdpi.com/2072-6643/15/14/3185 (accessed on 1 September 2023). This publication focuses on presenting the results regarding complementary feeding methods and the prevalence of food neophobia.

The study aimed to assess the prevalence of food neophobia among children aged 2–7 years, taking into account the method of complementary feeding, the length of breastfeeding, exclusive breastfeeding, the period of introduction of complementary foods, and the use of the BLW method during the period of dietary expansion.

## 2. Materials and Methods

### 2.1. Course of the Study

The study was conducted by questionnaire method, using an indirect survey technique: a web-based form computer-assisted web interview (CAWI). The questionnaire was disseminated to nurseries and kindergartens, which were randomly selected. The survey used closed groups of children and parents in nurseries and pre-schools/kindergartens. The survey was distributed through an instant messaging service for parents/carers to communicate with educational establishments. Parents affiliated with associations of parents of children aged 2–7 in different cities and provinces in Poland were also invited to participate in the survey. Considering the selection of the study group, we used the random method; the study sample selection was entirely random. We included inclusion and exclusion criteria in the study. Based on data from the Polish Ministry of Education and Science as of 30 September 2020, 90.1% of children aged 3–7 participated in various forms of preschool education (kindergarten). In the school year 2020/21, 22.4 thousand preschool education establishments were in operation. Three kindergartens were drawn from each voivodeship and invited to participate in the study; the database of kindergartens https://przedszkola.edubaza.pl/ (accessed on 1 September 2023) was used. The selection of nurseries, in turn, was based on a random selection of two nurseries from each of the voivodeships in Poland from the list of nursery school registers of the Ministry of Family and Social Policy. In these kindergartens and nurseries, groups of kindergartens/nurseries were drawn to which survey questionnaires were sent out using the CAWI method; in the nurseries, only groups of children over 2 years old were selected. All the study participants were informed of the purpose of the study. The parents surveyed were informed of the voluntary nature of their participation in the study, that anonymity would be maintained, and the rules for sharing data. The parents/guardians of the children who accepted the rules above for participation in the study were invited to participate in the further part of the study.

Adults whose children were of nursery or preschool age (mothers of children of nursery and preschool age) participated in the study. The period in which we conducted the study above was January–March 2023.

### 2.2. Selection of the Study Group

During the data analysis, we identified and verified the study group of parents. We observed that most participants in our study were mothers; only one father participated. Our research shows that mothers are the ones who most frequently contact the educational institution (nursery, kindergarten) through closed groups on instant messaging. At the same time, we also observed that mothers are responsible for feeding their children during the complementary feeding period, which may be due to the frequency of taking annual maternity/parental leave among women in Poland. Therefore, only mothers were enrolled in the study on the prevalence of feeding difficulties, including food neophobia at the age of 2–7 years, feeding during infancy, and feeding during complementary feeding. 

### 2.3. Eligibility Criteria

The study established inclusion and exclusion criteria. One of the primary inclusion criteria was that mothers gave informed consent to participate in the study. Once this consent was obtained, a questionnaire was made available to the mothers. 

The criteria for group selection included the following characteristics of the mothers: being of legal age, having at least one child aged 2–7 years, and having no formal knowledge of proper child nutrition and behavioural determinants of nutrition (education or profession related to nutrition, treatment, and education of children and adolescents).

The inclusion criteria for the study proper were as follows: being the mother/legal guardian of a child of nursery/pre-school age, i.e., 2 to 7 years, obtaining consent to participate in the study, and wholly and correctly completing the questionnaire used in the study. On the other hand, the criteria for exclusion from the study were the following: lack of mother’s consent to participate in the study, incorrectly completed or incomplete questionnaire, and child’s age below 2 years or above 7 years, as well as an existing disease in the child, which determines a specific method of feeding, e.g., diabetes, metabolic diseases, e.g., phenylketonuria, coeliac disease, food allergies and intolerances, autism spectrum disorders, recently undergone operations within the gastrointestinal tract of the child, or feeding with the use of an intragastric/intraluminal tube. This criterion was verified by assessing the answer to the question, “Does the child have any diseases, including chronic diseases?”. After considering all the inclusion and exclusion criteria, 585 pairs of mothers and their children were included in the final analysis.

The study was conducted by the ethical requirements for this type of research, considering the Declaration of Helsinki and the Polish law, i.e., the “Act on the Profession of Physician and Dentist”. In addition, an opinion was obtained from the Bioethics Committee of the Silesian Medical University in Katowice on conducting research in the Human Nutrition Department entitled “Food neophobia among infants and children”. The opinion on the research above was positive (BNW/NWN/0052/KB/34/23).

### 2.4. Research Tool

The study used an anonymous survey questionnaire consisting of five parts as the research tool. The first part of the research tool was about the metric data of the mothers and fathers/carer and their child. These data included information such as the child’s age and sex, birth (natural delivery, caesarean section), and current weight and height. Past medical history, including chronic diseases, feeding through a tube or fistula, or diseases determining a specific diet, as well as food allergies and intolerances were also considered. In the metric part, the data were obtained from the parents/legal guardians of the child: gender, age of the examined parent/legal guardian, socioeconomic data, place of residence, and mother’s or father’s or legal guardian’s education.

Some of the information obtained in the study was used from the “Child Health Booklet” (Polish—“Książeczka Zdrowia Dziecka”), which, according to the law in force in Poland, contains medical information concerning the child. This document contains information from, among other things, the child’s prenatal period, type of birth (natural delivery/caesarean section), and health status at birth (ABGAR scale). The “Child Health Booklet” also includes a description of patronage visits to the paediatrician; preventive examinations carried out, including dental examinations and immunizations performed; the history of occurrence of infectious diseases; and the occurrence of allergies and anaphylactic reactions, as well as radiological procedures and the provision of medical devices. In addition, there is information on sports exemption and information relevant to assessing the child’s normal development from birth, such as weight measurement and length/growth up to age 18. All entries in the “Child Health Booklet” are made by qualified medical staff, including a doctor, midwife, nurse, or other medical professional. The information is entered into the Health Booklet after the health service has been provided and, where this is not possible, is completed at the next visit based on individual internal records [35].

Based on the child’s age, weight, and length/height obtained from the “Child Health Booklet”, the average body weight was determined. Centile grids and 3 SD BMI for girls and boys aged 0–3 years from the WHO standard were used to assess the body weight of children up to 3 years of age. The children’s body weight was assessed and graded as underweight, normal weight, overweight, and obese. On the other hand, for children aged 3–7 years, the developmental norms for girls/boys aged 3–18 years, according to the OLAF and OLA studies, were used [36,37]. 

Another part of the questionnaire used in the study looked at feeding during infancy. The study included breast milk feeding, breast milk-only feeding, length of breast milk feeding, and the timing and method of CF (when CF introduction started; consistency of meals during CF, such as papes, pureed meals, finger-ready meals; products given to the child as CF; and the infant’s CF method, including the use of the BLW method). 

The third part of the study considered the child’s current diet. We included questions related to the child’s ability to use cutlery and the child’s food preferences and daily choices. We also considered taste preferences (bitter or sweet taste in meals), specific eating behaviours, and feeding disorders, including food neophobia. The research tool was developed based on the current dietary recommendations for a group of young children and the complementary feeding method developed by PTGHiŻD [1] based on ESPHGAN recommendations [6], as well as information on complementary feeding, including the use of the baby-led weaning method, food selectivity, and food neophobia occurring during this period of the child’s life [10,11,12,13,14,15,38].

The final part of the questionnaire focused on the prevalence of food neophobia. A standardised questionnaire assessing food neophobia among the children was used to assess food neophobia: Food Neophobia Scale—Children (FNSC) [39]. 

For the Food Neophobia Scale (FNS) questionnaire, we used the 10 items of the original FNS developed by Pliner and Hobden [39], back-translated. As the FNSC refers to children, the questions were worded by adding the prefix “my child”—adapted by Wardle, Carnell, and Cooke [40,41]. Moreover, in the remainder of this paper, we use the abbreviation FNSC. We used the following assessments: my child tries new and different foods all the time; my child does not trust new foods; if my child does not know what is in a particular food, he or she will not try it; my child likes foods from different countries; my child finds regional foods too strange to eat; my child tries new foods; my child is afraid to eat things that he or she has never eaten before; my child is very picky about the food we eat; my child will eat almost anything; my child would like to eat foods from other regions of Poland or other countries. In the FNSC, each item was rated on a 7-point agreement scale, from 1 = strongly disagree to 7 = strongly agree. All the food neophilia statements were reversed so that the scores indicated food neophobia. The total FNSC score was used to assess a person’s food neophobia level and propensity to try unfamiliar foods [39,40]. The interpretation of the results was based on the study “Prevalence of food neophobia in pre-school children from southern Poland and its association with eating habits, dietary intake and anthropometric parameters: a cross-sectional study” by Agnieszka Kozioł-Kozakowska, 2018 [42]. Thus, a score below 27 indicated a low risk of neophobia, 28–40 an intermediate risk of neophobia, and a score above 41 was considered high-risk. The study group of children was divided into 3 groups: a group with a low risk of food neophobia (referred to hereafter as 1—low neophobia), a group with a medium risk of food neophobia (2—medium neophobia), and a group with a high risk of food neophobia (3—high neophobia) [42].

### 2.5. Statistical Analysis

We used the following programmes to analyse the collected data: Microsoft Office Word 365 and Microsoft Office Excel 365. On the other hand, we performed the statistical analysis using Statistica v. 13.3. To characterise the data, we used the arithmetic mean and standard deviation (X ± SD) and the range of minimum and maximum values (min–max) in the study group. Moreover, for the statistical analysis, we used statistical tests, which were performed in Statistica v. 13.3. (StatSoft Inc., Tulsa, OK, USA). For the non-parametric characteristics and bivariate tables, Pearson’s Chi^2^ test was used to compare the children in the three groups: 1—low neophobia, 2—medium, and 3—high neophobia. The level of statistical significance adopted in the study was set at *p* ≤ 0.05. The Kruskal–Wallis test with a post hoc test was used to compare multiple independent groups.

## 3. Results

Table 1 shows the characteristics of the study group of children broken down by the risk of food neophobia. In the study group, according to the FNSC, 171 children (29.23%) had a low risk of food neophobia, 182 children (31.11%) had a medium risk of neophobia, and 232 children (39.66%) had a high risk of neophobia. There was no difference between the incidence of neophobia according to the sex of the child (*p* = 0.907), place of residence (*p* = 0.907), weight of the child (*p* = 0.095), type of delivery (*p* = 0.714), date of delivery (*p* = 0.601), and mother’s education (*p* = 0.532).

A correlation was observed between the age and risk of food neophobia (*p* = 0.0002). Statistically significant differences were found between children aged 2 and 4 (*p* = 0.003) and children aged 2 and 5 years (*p* = 0.049).

Table 2 shows the results for the total length of breast milk feeding in the study group of children. No statistically significant relationship was observed between the length of breast milk feeding and the incidence of risk of food neophobia; however, it was observed that the majority of the children studied were those fed with breast milk for longer than 6 months (314 children, 53.67%), of which 6–12 months accounted for 97 children (16.58%), 13–24 months accounted for 136 children (923.25%), and more than 24 months accounted for 81 children (13.85%). It should also be noted that the number of children not fed breast milk until 6 months of age was high, i.e., 249 children (42.56%). It should also be noted that the WHO recommends breastfeeding for more than 2 years; therefore, only 13.85% met the WHO assumptions.

Table 3 considers the length of EXCLUSIVE breastmilk feeding in the study group, considering the presence of food neophobia using FNSC. Exclusive breastfeeding means not giving milk formula to the child. The child consumes breast milk exclusively. No correlation was observed between the length of exclusive breastfeeding and the risk of food neophobia (*p* = 0.064). On the other hand, it was observed that 70 children (11.97%) were not exclusively fed with breast milk or not fed breastmilk at all. On the other hand, according to WHO recommendations, 248 children (42.39%) were exclusively fed breast milk until 6 months of age, while the rest had milk formula introduced: 122 children (20.85%) were fed breast milk for less than a month.

Table 4 shows the results for the initiation period of complementary feeding (CF) in the study of children about the incidence of food neophobia using the FNSC. During the initiation of dietary expansion, they also had no statistically significant effect on the incidence of risk of food neophobia (*p* = 0.957).

The study also looked at supplemental feeding of the child by feeding puree meals (e.g., carrot puree) and puree with lumps (e.g., rice with carrot puree). There were no significant correlations between the administration of puree (*p* = 0.095) and puree with lumps (*p* = 0.749) meals and the risk of food neophobia. Children who were given purees had a higher risk of food neophobia than those who were not (41.55% vs. 28.05%) (Table 5).

In analysing the data obtained during our study, we considered one of the complementary feeding methods: the baby-led weaning (BLW) method. In our study, according to the definition of the BLW method, we considered that if a child ate completely independently or mostly independently, the BLW method was applied to him. The BLW method was also applied to children who were occasionally spoon-fed by an adult—about 10% of feedings by the parent and 90% by the child eating independently.

The BLW method can be introduced when the child can sit up independently, around 6–7 months of age. In this case, it is based on skipping the spoon-feeding stage during complementary feeding and skipping pureed (mushy) foods, such as purees and purees with lumps. In the BLW method, various foods are given to the baby in solid form so that he can quickly grasp them independently. These foods can be, for example, sliced vegetables or fruits, such as bell peppers, cucumbers, carrots, pieces of broccoli/cauliflower, mango, melon, pear, peach, apple, sliced bread or rolls, pieces of meat, or pasta of different colours and shapes [6,7].

There was no statistically significant relationship between the use of the BLW method and the occurrence of the risk of food neophobia (*p* = 0.072).

A significant finding seems to be that children whose parents observed difficulties with introducing new foods during CF were at higher risk of food neophobia. We also included recommendations for the child to decide what he will eat during CF and how much he will eat. Neither the information regarding what the child will eat (*p* = 0.466) nor how much the child will eat (*p* = 0.059) correlated with the incidence of food neophobia risk.

Our study analysed the problems faced by parents during complementary feeding: vomiting reflex, spitting food out of the mouth, gagging, choking, and needing medical intervention. We observed no correlation between gagging (*p* = 0.88557), choking (*p* = 0.17597), and needing medical intervention (*p* = 0.61427) and the risk of associated neophobia. In contrast, we observed a correlation between the vomiting reflex and spitting food out of the mouth and a higher risk of food-related neophobia: *p* = 0.0195 and *p* = 0.03224, respectively (Table 6).

Table 7 analyses when the food product was first given to the child in the study group. The analysis also considered the presence of food neophobia using FNSC (*n* = 585). No correlation was observed between the presence of risk of food neophobia and the time of introduction of sugar (*p* = 0.286), salt (*p* = 0.063), and cow’s milk (*p* = 0.906) in the children’s CF; however, a correlation was observed between the time of introduction of nuts and the occurrence of the risk of food neophobia (*p* = 0.0003). The difference is related to the administration of nuts between 6 and 12 months of age and after 24 months of age (*p* = 0.011603). The earlier the children were given nuts, the lower their risk of food neophobia.

## 4. Discussion

Research indicates that breastfeeding positively affects the acceptance of new, unfamiliar foods in infants. It has also been hypothesised that the timing of the initiation of complementary feeding or the introduction of more textured foods may be necessary for the later acceptance of different food tastes and textures [41,43]. In a study by Coulthard et al., children who were introduced to lumpy foods at 10 months were more likely to have feeding difficulties, consume inadequate amounts of food, and be fussy eaters and refuse to eat at seven years of age compared to children who were introduced to pureed foods with lumps at the recommended time [44]. In a Dutch study by de Barse et al., children who had never been breastfed did not differ in the frequency of fussy eating from children who had been breastfed for six months or longer. It was observed in this study that children who had been breastfed for less than two months had a 0.70-point higher score on the “picky eating” scale than children who had been breastfed for 6 months or longer [45]. In a Brazilian study by Maranhao et al., the proportion of children with feeding difficulties between the ages of 2 and 6 years was comparable in the group exclusively breastfed for 6 months or less and in the group of children breastfed for more than 6 months [46]. In a U.S. study among children, no significant difference in weaning age was observed between choosy and non-choosy children at 5.5 years [47]. In a Turkish cross-sectional study by Orün E et al., the prevalence of problematic feeding behaviour in children aged 1 to 5 years did not differ significantly between the length of breastfeeding, with up to 39% of the children studied being characterised as picky eaters [48]. A study by Emmett et al. observed that the most vital factor predisposing a child to be a picky eater at 3 years and 2 months was the late introduction of lumpy foods into the infant’s diet (81% more likely). The child’s rejection of solid foods before 6 months of age (63%), feeding the child on demand (44%), and the mother indicating that feeding the child at 6 months was difficult for her (33%) were also shown to predispose a child to being a picky eater [49]. A Danish study evaluating the duration of exclusive breastfeeding was associated with a lower risk of picky eating behaviour at 2 to 6 years of age in children fed exclusively breast milk for 4–5 months than children fed only breast milk until the first month of life. In this study, the parents’ subjective evaluation assessed the child’s fussy eating behaviour [50]. In a review of the literature, Harris and Coultard indicated that breastfeeding is not necessary for children to be universally accepting of foods. In contrast, the authors emphasise that it is necessary to systematically and frequently introduce solid foods of different tastes and textures during CF [51]. In a meta-analysis by Bąbik et al., the authors highlighted that there is no substantial evidence to support the hypothesis that early feeding practices, namely, the timing of breastfeeding, the timing of the introduction of complementary feeding, or the choice of feeding technique, contribute to parent-reported feeding difficulties in children over 1 year of age [16]. A review of the literature by Mudholkar et al. [52] also pointed out that insufficient scientific evidence supports which complementary feeding techniques should be used to prevent children from becoming food choosy at a later stage of life. At the same time, both Bąbik and Mudholkar emphasised that in terms of the study of how children are fed in infancy and the occurrence of later food choosiness, including food neophobia, scientific research based on more rigorous testing methods should be conducted [16,52]. The present study also confirms the literature review by Bąbik et al. and Mudholkar et al. that the timing of breastfeeding, the timing of exclusive breastfeeding, the timing of dietary expansion, and the method of dietary expansion do not significantly affect the incidence of food neophobia [16,52].

Food neophobia in children may be associated with food choosiness and reluctance to try unfamiliar foods and dishes, resulting in nutrient deficiencies in the child’s diet. The rejection of unfamiliar, new foods reduces dietary diversity and makes it challenging to balance a child’s daily diet [52]; therefore, the present study aimed to investigate the prevalence of food neophobia in preschool children and the correlation between age, gender, infant feeding patterns, including complementary feeding, and the prevalence of feeding difficulties in the study group of children. Some studies indicate that 10 to 15 positive experiences with a new food are enough to accept it [53,54,55]. It has also been shown that children are more likely to try a new food if they see an adult or peer eating it [56]. Eating has a social dimension influenced by social and environmental factors that can lead to food neophobia. For example, parent/guardian characteristics significantly influence food neophobia in children. Food neophobia in children is positively correlated with parental food neophobia and negatively correlated with socioeconomic status and educational level [57,58]. Moreover, this is a topic where research should be expanded. 

According to the observations of Moding J. et al. conducted among children at the neonatal age, at 4, 6, 12, and 18 months, and then at 4.5 years of age, approach/withdrawal behaviours and the emotions associated with these behaviours will be associated with food neophobia. This study shows that some children with high levels of negative affect when confronted with novelty are likelier to develop food neophobia than their peers. This study indicates that food neophobia is associated with a concomitant tendency to withdraw in response to novelty. In the case of food neophobia, fear may motivate the avoidance of potential dangers associated with the consumption of harmful substances or toxins. Mothers whose children had high levels of negative feeding behaviour had higher rates of food neophobia. In addition, maternal pressure may further reinforce food neophobia at a later stage [27]. Our study confirms the research of Moding et al., in which children who had a vomiting reflex during CF and spit food out more often later in life had a higher risk of neophobia. The problems associated with introducing new foods into the diet during CF also determined later in life that they had a higher risk of neophobia, which suggests that temperamental traits play a role in the actual occurrence of neophobia; however, further research would be needed in this regard.

Perry et al. point out that health professionals, such as dietitians and paediatricians, should play an essential role in educating parents to understand neophobia as a normal developmental stage, but also teach them how to manage this behaviour through repeated neutral exposure, whereby parents can encourage their child to try (and eventually accept) new foods. The earlier the nutritional intervention, the sooner correct eating habits can be established in the child that will continue into adulthood [21]. Therefore, it is of the utmost importance to educate those raising children to shape their eating habits and eating behaviours, observe food neophobia and food selectivity, and guide them with appropriate nutrition education.

## 5. Conclusions

In the study group of children, the highest risk of food neophobia was characterized by children aged 4, 5, and 7 years. The length of breastfeeding and exclusive breastfeeding did not affect the risk of food neophobia. In the month in which CF was introduced, the children were fed using the BLW method, and introducing puree and puree with lump food into the children’s diet also did not affect the risk of food neophobia. It was shown, however, that children whose mothers observed difficulties during CF and whose children had a vomiting reflex and spat food out of their mouths during CF were more likely to develop food neophobia at the preschool age, which may suggest that neophobic behaviour has a biological basis.

In addition, summarising the results obtained in Part I of the study on the occurrence of feeding difficulties [34], it should be taken into account that breast milk feeding and the duration of CF in the study group did not affect the risk of feeding problems. Using the full BLW method during CF may protect the child from future feeding problems, such as food selectivity or picky eating. In our study, children with difficulties during CF, mainly vomiting reflexes, were more likely to experience feeding problems, such as food neophobia. Considering the conclusions of this publication and those of Part I of the study [49], attention should be paid to vomiting reflexes and spitting food out of the mouth during CF, as they may predispose children to later food selectivity and food neophobia. Considering alternative CF methods, such as BLISS/BLW, may prevent the occurrence of feeding difficulties, and it also seems essential to verify the individual child’s readiness for CF.

## 6. Strengths and Limitations of the Study

The results of our study should be interpreted with its limitations in mind. 

Some risk of error should be expected in the study due to the participants’ more significant interest in their children’s diet than in the general population. Our retrospective study may affect the false memory effect, especially in the group of mothers of older children, especially 5–7-year-olds. Their knowledge of their child’s infancy and complementary feeding may have been misremembered. 

The survey was conducted using the CAWI method, which can be criticised for lacking insight into the data collection process. However, our survey included mothers and their children from specific preschool and nursery groups. In addition, the parents were given detailed instructions on how to answer the questions before completing the survey. It is worth noting that the CAWI method as a type of data collection is widely accepted and convenient for collecting a large amount of information in groups in different cities of Poland, especially those that are often difficult to reach. 

The advantage of this survey is the group size: to date, most studies on feeding difficulties, such as food neophobia among children, have been conducted on smaller groups of respondents. It is also worth mentioning that very few studies have been conducted on this topic, especially in Poland, and the above study is another one we conducted. Moreover, no cross-sectional study has analysed the relationship between the use of the BLW method and difficulties during CF, and the CF method and the occurrence of food neophobia in the nursery and preschool groups.

## Figures and Tables

**Table 1:** Characteristics of the study group of mothers and their children at risk of food neophobia using the FNSC (*n* = 585).

	1—Low Neophobia		2—Medium Neophobia		3—High Neophobia		Total		*p* Value
	*n*	%	*n*	%	*n*	%	*n*	%	
Total	171	29.23%	182	31.11%	232	39.66%	585	100.00%	
Education (mother)									*p* = 0.532
primary school	5	35.71%	4	28.57%	5	35.71%	14	2.39%	
secondary school	44	29.93%	47	31.97%	56	38.10%	147	25.13%	
higher education/university	122	28.77%	131	30.90%	171	40.33%	424	72.48%	
Sex (children)									*p* = 0.907
boys	87	29.10%	91	30.43%	121	40.47%	299	51.11%	
girls	84	29.37%	91	31.82%	111	38.81%	286	48.89%	
Place of residence (mother and children):									*p* = 0.095
village	36	30.25%	34	28.57%	49	41.18%	119	20.34%	
city with up to 10,000 inhabitants	7	23.33%	11	36.67%	12	40.00%	30	5.13%	
city of 10–50,000 inhabitants	28	29.47%	35	36.84%	32	33.68%	95	16.24%	
city of 50–100,000 inhabitants	31	24.03%	33	25.58%	65	50.39%	129	22.05%	
city with more than 100,000 inhabitants	69	32.55%	69	32.55%	74	34.91%	212	36.24%	
Body weight (children):									*p* = 0.776
underweight	37	26.62%	44	31.65%	58	41.73%	139	23.76%	
normal weight	120	29.70%	129	31.93%	155	38.37%	404	69.06%	
overweight	12	38.71%	5	16.13%	14	45.16%	31	5.30%	
obesity	2	18.18%	4	36.36%	5	45.45%	11	1.88%	
Age (children):									*p* = 0.0002 *
2 years	56	43.08%	34	26.15%	40	30.77%	130	22.22%	
3 years	46	34.33%	45	33.58%	43	32.09%	134	22.91%	
4 years	14	15.38%	33	36.26%	44	48.35%	91	15.56%	
5 years	17	21.25%	26	32.50%	37	46.25%	80	13.68%	
6 years	18	21.95%	31	37.80%	33	40.24%	82	14.02%	
7 years	20	29.41%	13	19.12%	35	51.47%	68	11.62%	
Delivery (pregnancy week)									*p* = 0.601
29–31	1	25.00%	2	50.00%	1	25.00%	4	0.68%	
32–36	10	22.73%	13	29.55%	21	47.73%	44	7.52%	
after 37	160	29.80%	167	31.10%	210	39.11%	537	91.79%	
Type of birth									*p* = 0.714
naturally	93	29.62%	100	31.85%	121	38.54%	314	53.68%	
unplanned caesarean section	42	29.58%	45	31.69%	55	38.73%	142	24.27%	
planned caesarean section	36	27.91%	37	28.68%	56	43.41%	129	22.05%	

* Difference between 2- and 4-year-olds (*p* = 0.003), 2- and 5-year-olds (*p* = 0.049).

**Table 2:** Length of breast milk feeding in the study group of children with respect to risk of food neophobia using FNSC (*n* = 585) (*p* = 0.281).

Length of Time the Baby Has Been Fed Breast Milk	1—Low Neophobia		2—Medium Neophobia		3—High Neophobia		Total	
	*n*	%	*n*	%	*n*	%	*n*	%
was not fed with mother’s milk	6	20.69%	9	31.03%	14	48.28%	29	4.96%
less than 1 month	20	28.57%	24	34.29%	26	37.14%	70	11.97%
1–2 months	16	26.23%	17	27.87%	28	45.90%	61	10.43%
3–4 months	14	26.92%	15	28.85%	23	44.23%	52	8.89%
5–6 months	7	18.92%	13	35.14%	17	45.95%	37	6.32%
6–12 months	38	39.18%	29	29.90%	30	30.93%	97	16.58%
13–24 months	43	31.62%	43	31.62%	50	36.76%	136	23.25%
over 24 months	19	23.46%	28	34.57%	34	41.98%	81	13.85%
I am still feeding	6	31.58%	4	21.05%	9	47.37%	19	3.25%
I do not remember	2	66.67%	0	0.00%	1	33.33%	3	0.51%

**Table 3:** Length of exclusive feeding (exclusive breastfeeding means not giving modified milk to the baby; the child consumes only breast milk) breast milk in the study group with the presence of food neophobia using FNSC (*n* = 585) (*p* = 0.064).

Period during which the Child Has Been Fed ONLY Breast Milk	1—Low Neophobia		2—Medium Neophobia		3—High Neophobia		Total	
	*n*	%	*n*	%	*n*	%	*n*	%
was not fed with mother’s milk	13	26.53%	15	30.61%	21	42.86%	49	8.38%
was not fed exclusively with mother’s milk	9	42.86%	4	19.05%	8	38.10%	21	3.59%
less than 1 month	26	21.31%	38	31.15%	58	47.54%	122	20.85%
up to 2 months	9	32.14%	11	39.29%	8	28.57%	28	4.79%
up to 3 months	7	30.43%	6	26.09%	10	43.48%	23	3.93%
up to 4 months	7	23.33%	8	26.67%	15	50.00%	30	5.13%
up to 5 months	14	29.79%	14	29.79%	19	40.43%	47	8.03%
up to 6 months	76	30.65%	82	33.06%	90	36.29%	248	42.39%
I do not remember	10	58.82%	4	23.53%	3	17.65%	17	2.91%

**Table 4:** Timing of initiation of complementary feeding in study children by the presence of food neophobia using FNSC (*n* = 585) (*p* = 0.957).

Start Period for Complementary Feeding	1—Low Neophobia		2—Medium Neophobia		3—High Neophobia		Total
	*n*	%	*n*	%	*n*	%	*n*
before the child is 4 months old	8	40.00%	4	20.00%	8	40.00%	20
between 4 and 6 months of age	78	29.10%	82	30.60%	108	40.30%	268
after the baby is 6 months old	85	29.11%	92	31.51%	115	39.38%	292
I do not remember	0	0.00%	4	80.00%	1	20.00%	5

**Table 5:** Mode of complementary feeding in study children considering the presence of food neophobia using FNSC (*n* = 585).

	1—Low Neophobia		2—Medium Neophobia		3—High Neophobia		Total		*p* Value
	*n*	%	*n*	%	*n*	%	*n*	%	
Method of complementary feeding:									
Puree:									*p* = 0.095
yes	142	28.23%	152	30.22%	209	41.55%	503	85.98%	
not	29	35.37%	30	36.59%	23	28.05%	82	14.02%	
Puree with lumps:									*p* = 0.749
yes	122	29.19%	124	29.67%	172	41.15%	418	71.45%	
not	49	29.34%	58	34.73%	60	35.93%	167	28.55%	
Using the BLW method during CF *:									
no BLW	43	27.92%	42	27.27%	69	44.81%	154	26.32%	*p* = 0.072
half BLW	93	27.19%	112	32.75%	137	40.06%	342	58.46%	
BLW	3	27.27%	7	63.64%	1	9.09%	11	1.88%	
full BLW	32	41.03%	21	26.92%	25	32.05%	78	13.33%	
Difficulties in introducing new foods in the child:									
I do not remember	2	6.06%	10	30.30%	21	63.64%	33	5.64%	*p* = 0.000
There were no problems during CF with the baby’s diet.	153	37.05%	133	32.20%	127	30.75%	413	70.60%	
There were problems in during CF with the child’s diet.	16	11.51%	39	28.06%	84	60.43%	139	23.76%	
The child decided on his/her own WHAT to eat during CF									
always	99	30.56%	102	31.48%	123	37.96%	324	55.38%	
sometimes	49	27.07%	53	29.28%	79	43.65%	181	30.94%	*p* = 0.466
never	23	28.75%	27	33.75%	30	37.50%	80	13.68%	
The child decided himself/herself how much to eat during CF									
always	160	31.07%	157	30.49%	198	38.45%	515	88.03%	
sometimes	9	18.00%	16	32.00%	25	50.00%	50	8.55%	*p* = 0.059
never	2	10.00%	9	45.00%	9	45.00%	20	3.42%	

* No BLW—child entirely or mostly spoon-fed by an adult. Half BLW—baby half fed by an adult with a spoon, half eating independently. BLW—child occasionally spoon-fed by an adult (approximately 10 feedings of 90 on their own). Full BLW—the child ate completely or mostly independently.

**Table 6:** Prevalence of problems during complementary feeding in the study group of children, taking into account the presence of food neophobia using the FNSC (*n* = 585).

Problems during CF		1—Low Neophobia		2—Medium Neophobia		3—High Neophobia		Total		
		*n*	%	*n*	%	*n*	%	*n*	%	*p* Value *
Vomiting reflex	yes	37	22.56%	48	29.27%	79	48.17%	164	28.03%	*p* = 0.0194 Vcr = 0.1160676
Spat food out of its mouth	yes	94	25.47%	122	33.06%	153	41.46%	369	63.08%	*p* = 0.03224 Vcr = 0.1083587
Gagging	yes	56	30.60%	56	30.60%	71	38.80%	56	30.60%	*p* = 0.88557
Choking	yes	7	16.67%	15	35.71%	20	47.62%	42	7.18%	*p* = 0.17597
Choked and needed medical attention	yes	1	50.00%		0.00%	1	50.00%	2	0.34%	*p* = 0.61427

* Pearson’s Chi^2^ test with Cramer’s V.

**Table 7:** Time at which a food product was first given to a child in the study group, taking into account the presence of food neophobia using the FNSC (*n* = 585).

Food Product	1—Low Neophobia		2—Medium Neophobia		3—High Neophobia		Total		
	*n*	%	*n*	%	*n*	%	*n*	%	*p* Value
SUGAR									
not yet started	3	37.50%	2	25.00%	3	37.50%	8	1.37%	*p* = 0.286
between 6 and 12 months of age	36	26.09%	45	32.61%	57	41.30%	138	23.59%	
between 12 and 24 months	99	30.18%	96	29.27%	133	40.55%	328	56.07%	
after 24 months of age	29	32.58%	33	37.08%	27	30.34%	89	15.21%	
I do not remember	4	18.18%	6	27.27%	12	54.55%	22	3.76%	
SALT									
not yet started	3	50.00%	1	16.67%	2	33.33%	6	1.03%	*p* = 0.063
between 6 and 12 months of age	23	39.66%	20	34.48%	15	25.86%	58	9.91%	
between 12 and 24 months	95	30.06%	87	27.53%	134	42.41%	316	54.02%	
after 24 months of age	23	39.66%	20	34.48%	15	25.86%	58	9.91%	
I do not remember	3	13.64%	7	31.82%	12	54.55%	22	3.76%	
VARIOUS NUTS * (including PEANUT BUTTER, PEANUT FLOUR, etc.) *									
not yet started	13	28.26%	11	23.91%	22	47.83%	46	7.86%	*p* = 0.0003 **
between 6 and 12 months of age	54	42.52%	38	29.92%	35	27.56%	127	21.71%	
between 12 and 24 months	63	27.16%	81	34.91%	88	37.93%	232	39.66%	
after 24 months of age	34	24.64%	42	30.43%	62	44.93%	138	23.59%	
I do not remember	7	16.67%	10	23.81%	25	59.52%	42	7.18%	
COW’S MILK									
not yet started	3	15.00%	9	45.00%	8	40.00%	20	3.42%	*p* = 0.906
between 6 and 12 months of age	56	33.33%	44	26.19%	68	40.48%	168	28.72%	
between 12 and 24 months	85	28.81%	96	32.54%	114	38.64%	295	50.43%	
after 24 months of age	23	25.84%	30	33.71%	36	40.45%	89	15.21%	
I do not remember	4	30.77%	3	23.08%	6	46.15%	13	2.22%	

* E.g., peanuts, walnuts, cashews, hazelnuts. ** The difference between giving nuts between 6 and 12 months of age and after 24 months of age (*p* = 0.011603).

## Data Availability

The data presented in this study are available on request from the corresponding author. The data are not publicly available due to restrictions that apply to the availability of these data.
